# Loss of LIMCH1 predicts poor prognosis in patients with surgically resected Lung Adenocarcinoma: A study based on Immunohistochemical Analysis and Bioinformatics

**DOI:** 10.7150/jca.47883

**Published:** 2021-01-01

**Authors:** He Cao, Jing Zhao, Zhen Chen, Wenjia Sun, Kexin Ruan, Jianya Zhou, Jianying Zhou

**Affiliations:** 1 Department of Respiratory Disease, Thoracic Disease Center, The First Affiliated Hospital, College of Medicine, Zhejiang University, Hangzhou 310003, China.; 2 Department of Pathology, The First Affiliated Hospital, College of Medicine, Zhejiang University, Hangzhou 310003, China.

**Keywords:** lung adenocarcinoma, LIMCH1, TCGA, immunochemistry, prognosis

## Abstract

**Background:** LIMCH1, a novel actin-binding protein, is reported to correlate with tumorigenesis in multiple cancer types, but its clinical prognostic value in lung adenocarcinoma (LUAD) patients remains unclear.

**Methods:** A total of 196 patients with LUAD who underwent R0 resection were included for analysis. We integrated immunohistochemistry (IHC) and data mining analyses to determine LIMCH1 expression in tumor specimens; the chi-square test was used to explore the correlation between clinicopathologic factors and LIMCH1 expression in LUAD; Kaplan-Meier curves and the Cox proportional hazards model were used to investigate the clinical prognostic role of LIMCH1 expression in patients with LUAD; and DAVID enrichment and gene set enrichment analysis (GSEA) were used to determine the underlying molecular mechanism.

**Results:** LIMCH1 protein and mRNA expressions were significantly decreased in LUAD tissues. LIMCH1 mRNA expression was a potential diagnostic indicator in the TCGA cohort, and was associated with poor prognosis. IHC results in our LUAD cohort demonstrated that the LIMCH1 expression level was significantly associated with pleural invasion, tumor length, tumor differentiation grade, and clinical tumor stage. Patients with higher LIMCH1 expression had longer overall survival times. Cox multivariate survival analysis showed that LIMCH1 expression independently predicted the outcome. GO and KEGG clustering analyses showed that LIMCH1-related genes may be involved in 'cell adhesion', 'signal transduction', and several cancer-related pathways. GSEA showed 8 enriched hallmarks in the low LIMCH1 expression group, including mTOR signaling, MYC signaling, DNA repair, and G2M checkpoint.

**Conclusions:** Our findings suggest that LIMCH1 may serve as a promising biomarker to predict LUAD prognosis.

## Introduction

Lung cancer is one of the most common cancer types, and is a major threat to global health. Lung adenocarcinoma (LUAD) and lung squamous carcinoma (LUSC), the main histological forms of lung cancer, rank as the most aggressive malignancies. Patients with LUAD account for ~50% of all lung cancer cases, and the rate is continually increasing 1, 2. Based on cancer statistics, 1.8 million deaths worldwide are attributed to lung cancer, and LUAD accounts for ~40% of these cases 3. Rapid progress in molecular genetics has begun to reveal genetic mechanisms involved in lung cancer. Genetic factors have been shown to play a pivotal role in LUAD tumorigenesis. As a result of breakthroughs in molecular genetics, EGFR-tyrosine kinase inhibitors (TKIs) and PD-1/PD-L1 inhibitors have made significant breakthroughs in treating patients with LUAD. However, not all patients with LUAD are able to benefit from these treatments, and the survival rate is still unsatisfactory. Other reliable biomarkers are urgently needed.

Actin-binding proteins regulate various aspects of actin dynamics, and many actin dynamic- related genes have been reported to play critical roles in the development of tumors. Espin (ESPN), an actin-filament-binding protein, regulates organization, actin dynamics, and signaling transduction in the actin filament-rich cells. Li et al. reported that decreases in endogenous ESPN slowed esophageal squamous cell carcinoma (ESCC) cell growth and suggested ESPN as a novel therapeutic target 4. Twinfilin-1 (TWF1) is another conserved actin-binding protein, which is overexpressed in non-small-cell lung cancer (NSCLC), and is an independent predictor of poor outcomes in patients with LUAD 5.

LIMCH1 is located on chromosome 4p13 and encodes a novel actin-binding protein. It is expressed broadly in many human organs, including the lungs, spleen, heart, and brain. LIMCH1 could directly bind to non-muscle myosin II (NM-II) to regulate the NM-II activity, which is involved in cell movement. The depletion of LIMCH1 in HeLa cells attenuated the number of actin stress fibers and increased cell migration ability 6. Recent studies have reported its specific tumorigenesis role in several cancer types. Bersini et al. identified that low expression of LIMCH1 were associated with advanced tumor stages in patients with triple-negative breast cancer 7. In clear cell renal cell carcinoma (ccRCC), Eckel-Passow and colleagues found that LIMCH1 expression can increase the risk of smoking-related ccRCC 8. As for lung cancer, Liu et al. performed analyses in silicon and found downregulation of LIMCH1 mRNA expression in LUAD tissues compared with normal tissues 9. In another study, Zhang et al. confirmed that LIMCH1 mRNA expression was decreased in clinical NSCLC samples. Overexpression of LIMCH1 inhibited the growth of the A549 cell line 10. A previous study conducted by Karlsson et al. revealed a new biomarker of the paralogous protein LMO7 11. Nonetheless, the clinical prognostic value of LIMCH1 in patients with LUAD remains unclear.

Therefore, in this study, we used immunohistochemistry (IHC) to examine the prognostic relevance of LIMCH1 expression in our large LUAD cohort. We also explored the underlying molecular mechanisms by bioinformatics analysis.

## Methods

### Patients and Tissue Samples

**Figure 1:**
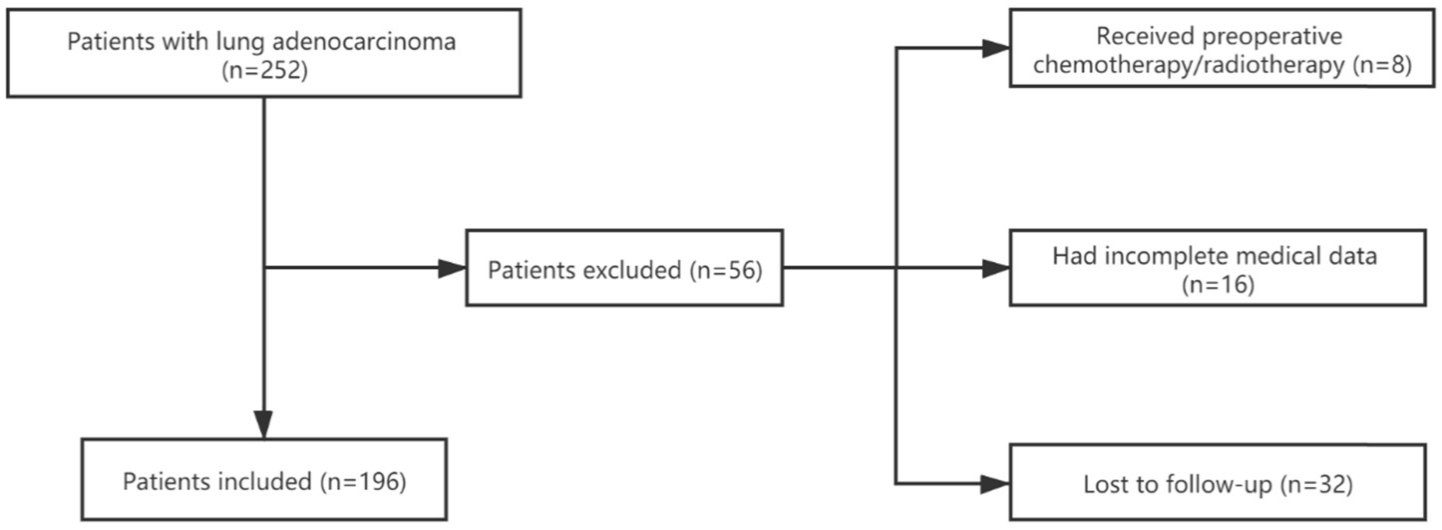
Flowchart of the enrollment process.

We retrospectively collected clinical data and tissue samples for 196 patients with LUAD who underwent R0 resection between January 2011 and March 2013 at the First Affiliated Hospital of Zhejiang University in Hangzhou, China. We also collected 9 matched adjacent normal tissues. We excluded patients who were lost to regular follow-up, had incomplete clinical information, or had ever received chemotherapy or radiotherapy before surgery (Figure 1). All clinical data in this study were collected from the hospital's electronic medical record system. The hospital's ethics committee approved the study, and informed consents had previously been provided before the index surgery.

All of the samples selected were pathologically confirmed as LUAD. Histological tumor grading and classifications were evaluated according to the World Health Organization's (WHO) grading system 12. Tumor data included clinical stage, location, length, and differentiation. Patient data included sex, age, smoking history, and length of survival. The last follow-up was in December 2019.

### Tissue Microarray and IHC Staining

A 3-mm-diameter core of a representative tumor area was selected to construct the tissue microarray (TMA). For IHC staining, TMA slides were de-paraffinized with xylene and then rehydrated with graded ethanol, after which antigens were retrieved. Endogenous peroxidase activity was reduced by saturating it with 3% hydrogen peroxide, and the slides were blocked in goat serum. After blocking, the slides were stained with the pre-diluted anti-LIMCH1 antibody (Abcam, ab96178). The next day, 3,3-diaminobenzidine (DAB) was used according to the manufacturer's instructions to stain the samples.

IHC results were inspected by 2 pathologists, who were blinded to the patient data. The LIMCH1 staining scores were semi-quantified using a modified immunoreactive scoring (IRS) system based on the staining intensity and percentage of epithelial cells within the tumor 13. Intensity was detected as no staining (0), weak (1), moderate (2), or strong (3). Immunoreactivity was detected as no staining (0), 1%-25% (1), 26%-50% (2), 51%-75% (3), or >75% (4); (see Figure 3E-L). The final IHC score was then calculated by multiplying the grades for the low (score < 7) and high (score ≥ 7) groups.

### LIMCH1 Expression in Public Datasets

We downloaded the expression profile and clinical information of the GDC TCGA-LUAD cohort from UCSC Xena (https://xena.ucsc.edu/) 14. Data from 524 primary tumors in patients with LUAD and 59 controls were obtained for RNA-seq analysis. Of these, who had a primary tumor, 511 cases had intact overall survival (OS) data. We extracted the demographic and clinical parameters for these patients. We also screened the expression of LIMCH1 between LUAD and normal groups by using Oncomine (https://www.oncomine.org/) 15. This online dataset provides access to the molecular profiling data for tissues and cells from many microarray chips. We also downloaded the LIMCH1 protein expression from the Human Protein Atlas (http://www.proteinatlas.org) 16, 17.

### Clustering Analysis of LIMCH1 Co-expressed Genes

To investigate the potential mechanisms responsible for LIMCH1 expression, we obtained LIMCH1-related co-expressed genes from the MEM (http://biit.cs.ut.ee/mem/index.cgi) 18 and cBioPortal databases (http://www.cbioportal.org) 19. We extracted the results from the top 5000 co-expressed genes in MEM. In cBioPortal, 20034 genes were co-expressed with LIMCH1 in LUAD (TCGA, Provisional). Of these, 1303 genes were included after being filtered with an absolute value of the Pearson's correlation coefficient >0.3. The intersection of the results from MEM and cBioPortal was then visualized using the Venny tool (http://bioinfogp.cnb.csic.es/tools/venny/index.html) 20. Next, the gene-annotation enrichment analysis and functional annotation clustering of these co-expressed genes were analyzed using the DAVID Bioinformatics Resources 6.8 database (https://david.ncifcrf.gov/) 21.

### Gene Set Enrichment Analysis

Gene Set Enrichment Analysis (GSEA) was performed by using GSEA v3.0 software from the Broad Institute, which computes 1000 permutations to find whether significant and concordant differences exist between 2 physiological states. Patients with LUAD were grouped by the median expression of LIMCH1, and h.all.v7.2.symbols.gmt was used as the reference gene set. A gene set is considered to be significantly enriched when a normal *P* value is <0.05, and a false discovery rate (FDR) is <0.25.

### Statistical Analysis

The data were analyzed using SPSS 22.0. The clinicopathological data were compared using the chi-squared test or the two-sided Student's t-test. Receiver operating characteristic (ROC) curves were used to assess diagnostic ability, the Kaplan-Meier method was used for overall survival analysis, and the log-rank test was conducted to examine the significance of the difference between survival curves. Univariate and multivariate analyses were performed to determine the prognostic value between different clinicopathological factors and survival. Statistical significance was set at *P*<0.05.

## Results

### Aberrant LIMCH1 down-regulation in TCGA and the clinical LUAD cohort

**Figure 2:**
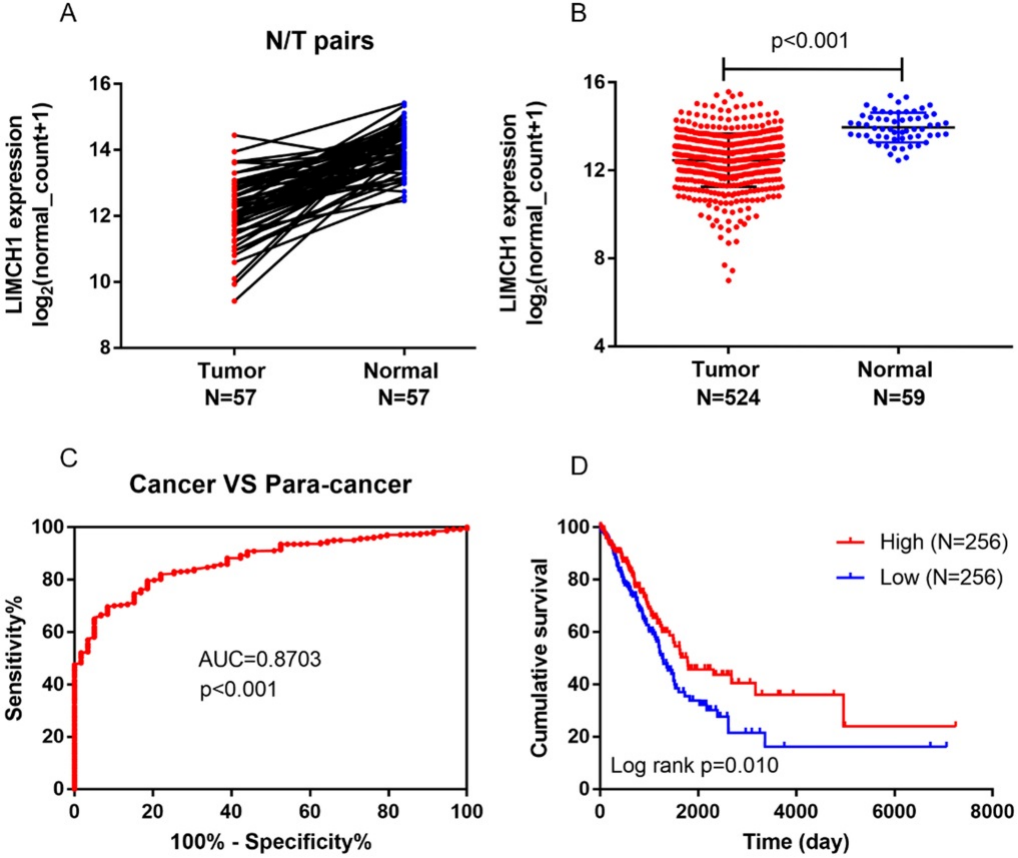
** Aberrant LIMCH1 expression, prognostic value, and survival analysis in the TCGA-LUAD cohort.** (**A**) Low expression of LIMCH1 in 57 paired of LUAD tissues. (**B**) LIMCH1 mRNA expression decreased in LUAD tissues than normal lung tissues. Diagnostic value (**C**) and survival analysis (**D**) of patients from the TCGA cohort.

We first determined the LIMCH1 mRNA expression level using TCGA data. As shown in Figure 2A-B, among 57 paired LUAD samples, decreased LIMHC1 expression was observed frequently in tumor tissues than in the adjacent non-tumorous tissues, the LIMHC1 level was consistently down-regulated in 524 samples of LUAD tissues compared with the 59 samples of normal lung tissues. We also observed a lower LIMCH1 level in a total of 7 GEO datasets from the Oncomine database (Table 1).

**Figure 3:**
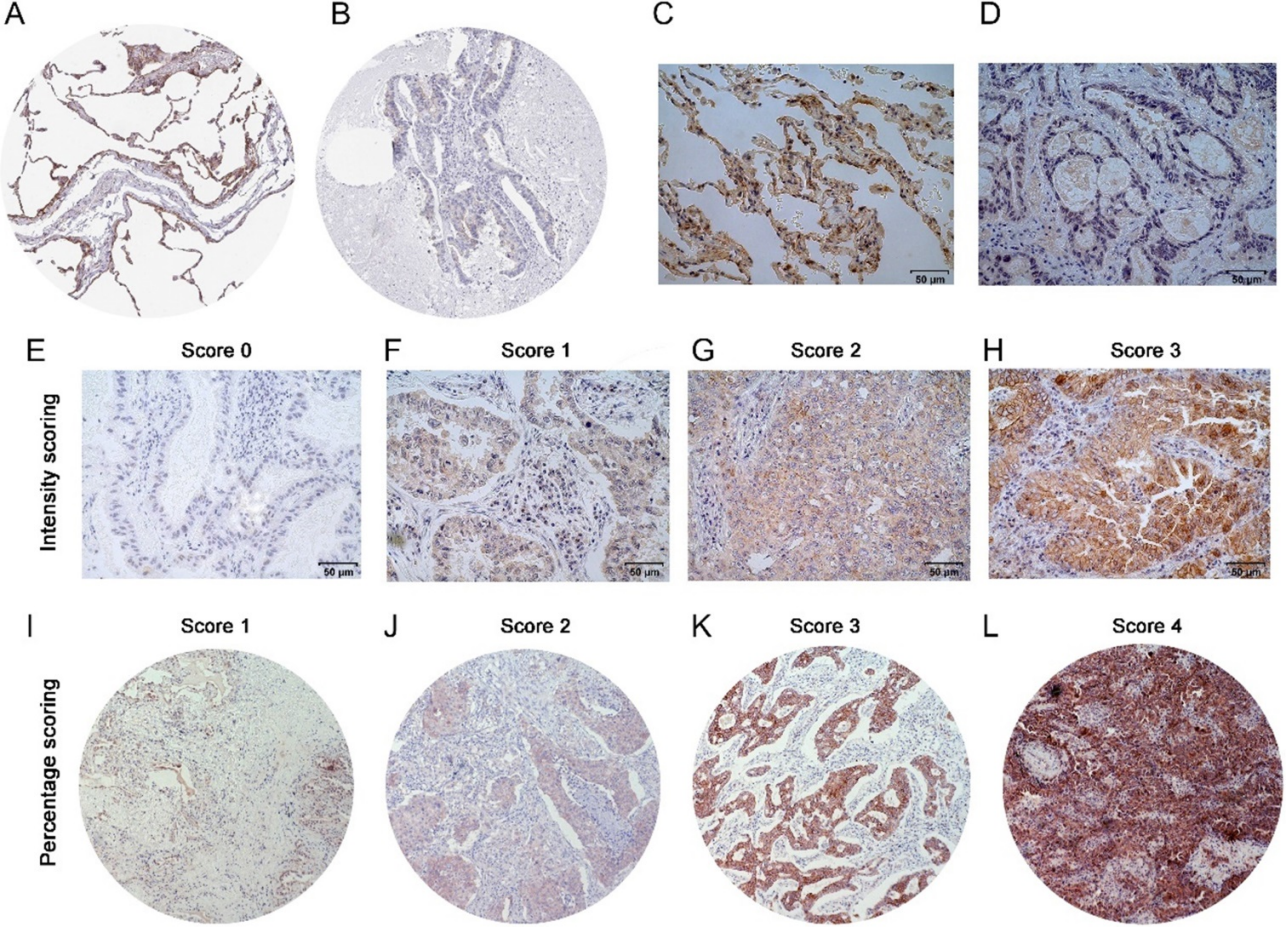
** The protein expression of LIMCH1 in LUAD tissues and non-tumor tissues. IHC staining of normal lung tissue** (**A**) and LUAD tissue (**B**) from HPA datasets. Representative images of IHC staining of LIMCH1 in paired adjacent normal tissues (**C**) and LUAD tissues (**D**) (Magnification ×400). Tumor tissues without LIMCH1 staining which score=0 (**E**); weak staining of LIMCH1 which score=1 (**F**); moderate staining of LIMCH1 which score=2 (**G**); strong staining of LIMCH1 which score=3 (**H**); (Magnification ×400). Representative images of step percentages of LIMCH1 staining in 1%-25% which score=1 (**I**); LIMCH1 staining percentage of 26%-50% which score=2 (**J**); LIMCH1 staining percentage of 51%-75% which score=3 (**K**), LIMCH1 staining percentage of >75% which score=4 (**L**); (Magnification ×40). Bar scale: 50 µm.

We performed IHC to test the protein levels of LIMCH1 in LUAD tissues. As shown in Figure 3, LIMCH1 was positively stained in the cytoplasmic, membranous, and perinuclear regions in the LUAD samples. In 9 paired LUAD tissues (Figure 3C-D), we observed a higher percentage of expression in adjacent normal tissues (7/9) than in the matched tumor tissues (2/9). Moreover, IHC evidence from the Human Protein Atlas database supported the downregulation of LIMCH1 protein in LUAD tissues (Figure 3A-B).

### Diagnostic and Prognostic Roles of LIMCH1

In the TCGA cohort, ROC curves showed that LIMCH1 expression was a powerful diagnostic indicator (Figure 2C, AUC=0.870, *P*<0.001). Survival analysis also showed that low LIMCH1 expression was associated with poor prognosis in patients with LUAD (Figure 2D). Multivariate Cox regression analysis (Table 2) showed that the T stage (HR 2.009, 95% confidence interval [CI] 1.364-2.958), N stage (HR 2.313, 95% CI 1.715-3.119), and LIMCH1 expression (HR 0.689, 95% CI 0.512-0.927) were significant independent prognostic predictors (*P*<0.05) for the LUAD cohort from TCGA.

### LIMCH1 Expression and Clinicopathological Factors

In our clinical cohort, the patients' age at surgery ranged from 35 to 87 years, with a median age of 61 years. Ninety-five patients (48.5%) were female, and 70 patients (35.7%) had a smoking history. One hundred twenty-five patients (63.8%) died during a median follow-up of 48 months (3-104 months). Patients were divided into 2 groups (low expression vs high expression) based on the IHC staining data. Lower LIMCH1 expression positively correlated with several parameters, including pleural invasion, tumor length, tumor differentiation grade, clinical tumor stage and therapeutic strategy (all *P*<0.05). However, no significant association was observed in age, gender, or smoking history between these 2 groups (Table 3).

### Survival Analysis

**Figure 4:**
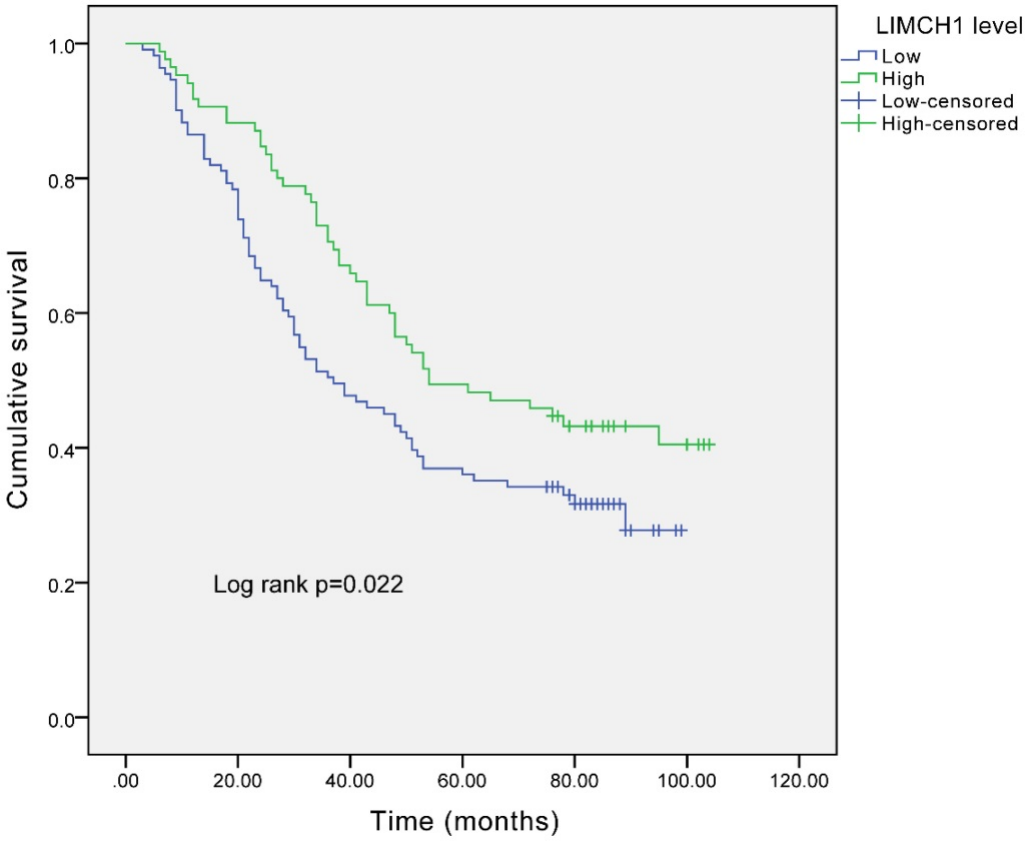
Survival analysis of LUAD patients with different expressions of LIMCH1 based on IHC staining.

The survival curves (Figure 4) demonstrated that the patients with higher LIMCH1 expression had better prognosis (*P* =0.022). We conducted univariate and multivariate analyses to examine the independent prognostic significance of LIMCH1 expression. The results of univariate analysis indicated that pleural invasion, differentiation grade, tumor length, T stage, N stage, TNM stage, and LIMCH1 expression were associated with overall survival in patients with LUAD, and multivariate analysis confirmed that LIMCH1 expression (HR 0.545, 95% CI 0.358-0.830), N stage (HR 3.618, 95% CI 1.281-10.217), and TNM stage (HR 3.662, 95% CI 2.261-5.931) were independent prognostic factors (Table 4).

### Bioinformatics Analyses

**Figure 5:**
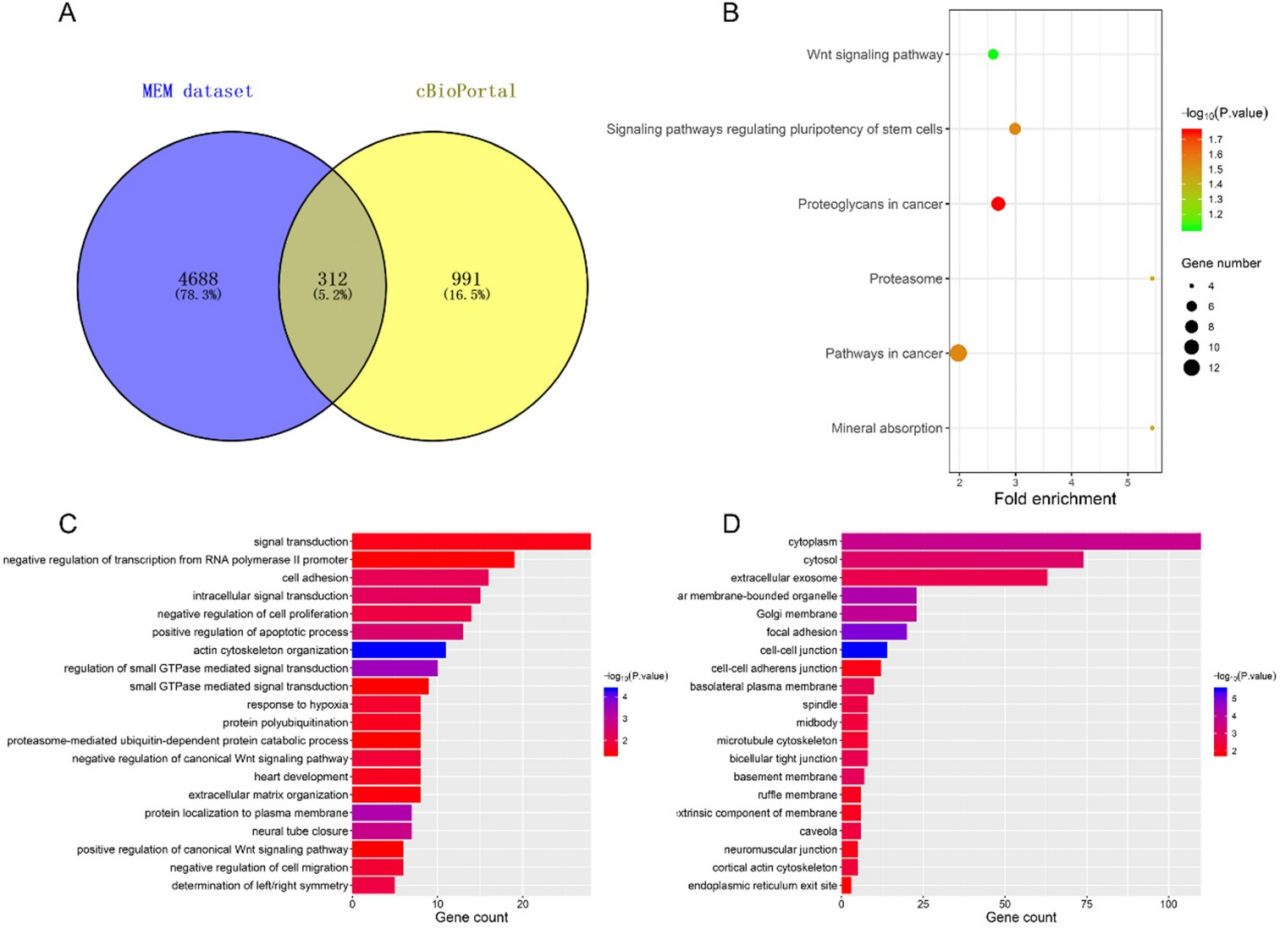
**DAVID analysis of co-expressed genes.** (**A**) the intersection of co-expressed genes between MEM and cBioPortal datasets. (**B**) KEGG analysis revealed significantly enriched pathways. GO terms of Biological Process (**C**) and Cellular Component (**D**).

We selected 312 co-expression genes contained in both the Affymetrix GeneChip Human Genome U133 Plus 2.0 (containing 1794 datasets) by MEM and the cBioPortal TCGA-LUAD dataset (provisional) for the bioinformatics analyses (Figure 5A). Functional annotation (GO and KEGG pathway) was then carried out using DAVID. The top 20 significant enrichment terms are shown in Figure 5C-D. These co-expressed genes were enriched in 'actin cytoskeleton organization', 'positive regulation of apoptotic process' and 'negative regulation of cell proliferation' in the biological processes; in the 'cell-cell junction', 'cytoplasm', and 'focal adhesion' in cellular components; in 'protein kinase binding' , 'actin binding', and 'GTPase activity' in the molecular functions. KEGG pathway analysis revealed enriched terms as 'proteoglycans in cancer', 'signaling pathways regulating pluripotency of stem cells', and 'Wnt signaling pathway' (Figure 5B), suggesting that those genes were involved in development of LUAD.

**Figure 6:**
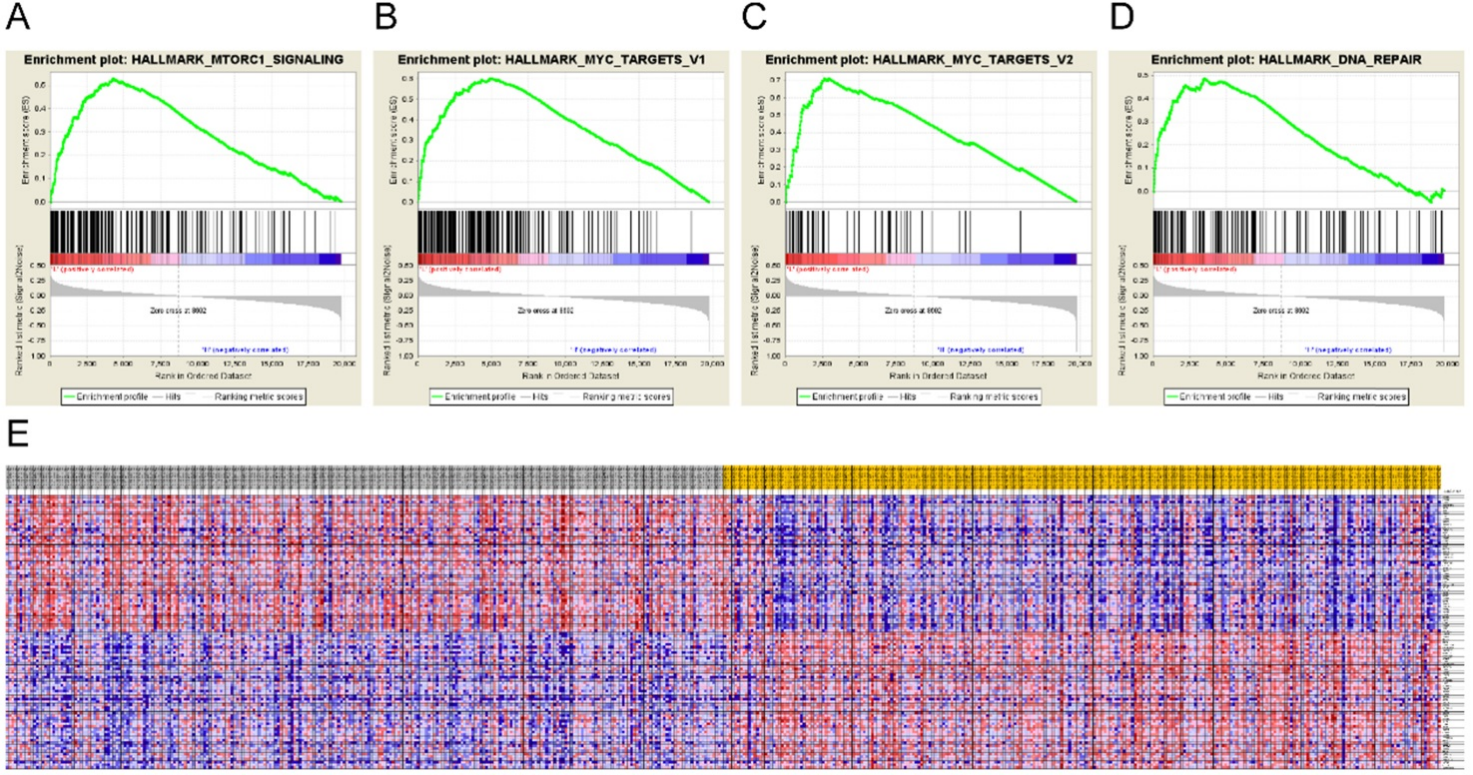
** Gene Set Enrichment Analysis of LIMCH1.** (**A-D**) The most significantly associated pathways. (**A**) MTORC1 signaling; (**B**) MYC targets; (**C**) DNA repair; (**D**) G2M checkpoint; (**E**) Heat Map of the top 100 genes.

To identify the mechanisms underlying LIMCH1 expression, we also conducted GSEA. Eight hallmarks were significantly enriched in the low LIMCH1 expression group, including mTORC1 signaling, MYC targets, DNA repair, and G2M checkpoint (Figure 6A-E and Table 5). These analyses indicated that LIMCH1 was closely associated with cell cycle and proliferation to affect LUAD.

## Discussion

LUAD is a highly malignant disease with various genetic backgrounds. Despite recent advances in precision medicine, the 5-year survival rate remains low. Therefore, novel prognosis biomarkers are urgently needed to improve the outcome of patients with LUAD. In this research, we demonstrated that LIMCH1 was downregulated in mRNA and protein levels in LUAD tissues. The low percentage of LIMCH1 staining was an independent risk factor for poor outcome in patients with LUAD. We also explored the possible mechanism for this finding.

LIMCH1, which also named KIAA1102, encodes actin stress-fibers associated protein. It promotes the phosphorylation of regulatory subunit MRLC/MYL9 to activate cell migration. Several recent studies reported that LIMCH1 expression was associated with breast cancer, clear cell renal cell carcinoma, and lung cancer. However, its clinical prognostic value in patients with LUAD remains unclear.

In this study, we first showed that LIMCH1 expression is downregulated in LUAD tissues by performing IHC staining in our cohort. Data mining from TCGA, Oncomine, and HPA datasets also supports our results. To our knowledge, this is the first study to discuss the protein expression level of LIMCH1 in LUAD. We found that LIMCH1 plays a role in tumorigenesis and tumor progression. Based on the strong evidence of differentiative expressions in tissues and of the actin stress binding function in specific cell types, we explored the prognostic role in patients with LUAD. Our results show that reduced LIMCH1 expression is associated with pleural invasion, more substantial tumor length, lower differentiation grade, and more advanced tumor stage. In both TCGA-LUAD and our LUAD datasets, patients with lower LIMCH1 expression have shorter survival times. Cox regression analysis revealed that LIMCH1 expression independently predicts poor overall survival.

A previous study of the molecular mechanisms involved in cancer development, using yeast 2-hybrid screen found that LRIG proteins interacted with LIMCH1 peptides 11. Zhang et al. reported that LIMCH1 was reciprocally co-immunoprecipitated with HUWE1 to ubiquitinated p53. Degradation of p53 affects downstream proteins to regulate cell proliferation 10. In our study, GO and KEGG analyses of co-expressed genes were conducted to identify the function of LIMCH1. Gene-annotation enrichment analysis showed that LIMCH1 co-expressed gene function is associated with cell adhesion and actin cytoskeleton organization, which is consistent with previous studies. KEGG analysis demonstrated that these genes are involved in several cancer-related pathways, and GSEA identified 8 hallmark pathways, including MTORC1, MYC targets, DNA repair, G2M checkpoint signaling. DNA repair and cell cycle seriously affected cell proliferation, which was associated with LUAD progression. Further studies are needed to verify these mechanisms *in vitro* and *in vivo*.

There are still certain limitations in this current study. First, the clinical LUAD cohort was obtained from our single center, and the clinical parameters were collected retrospectively. Thus, the selection bias could not be avoided. More prospective multi-center studies are needed to validate these results. Second, we selected just one core per tumor to construct the tissue microarray, thus, the tumor heterogeneity could be a challenge. Future studies should add more cores from each tumor block to eliminate the problem to a large part.

In conclusion, our current study demonstrated that LIMCH1 expression is associated with aggressive tumorigenesis, and that decreased expression of LIMCH1 predicts a shorter overall survival time, which is an independent prognostic factor in patients with LUAD. LIMCH1 appears to be a novel prognostic target for LUAD, but more real-world studies are needed to confirm the critical mechanisms underlying cancer development in patients with LUAD.

## Figures and Tables

**Table 1:** The relative expression of LIMCH1 between lung adenocarcinoma tissues and adjacent normal tissues in Oncomine database

GEO datasets	Normal	Tumor	*p* value	Fold change
GSE3398	6	39	5.05E-08	-4.318
GSE7670	30	27	3.02E-13	-3.791
GSE10072	49	58	3.33E-25	-3.336
GSE2514	19	20	7.88E-08	-3.363
GSE19188	65	45	5.20E-16	-4.087
GSE32863	58	58	1.94E-22	-2.968
GSE31210	20	226	1.01E-12	-2.177

**Table 2:** Multivariate Cox proportional hazards regression of overall survival in TCGA-LUAD cohort

Variables	HR	95% CI	*p* value
**T**			
T1-T2 vs T3-T4	2.009	1.364-2.958	<0.001
**N**			
N0 vs N1-3	2.313	1.715-3.119	<0.001
**M**			
M0 vs M1	1.681	0.956-2.953	0.071
LIMCH1 expression	0.689	0.512-0.927	0.014

**Table 3:** Correlations between LIMCH1 expression and clinical parameters of 196 patients with lung adenocarcinoma

Category	Case (N, %)	LIMCH1 expression		*p* value
	196 (100%)	Low (n=111)	High (n=85)	
**Age (years)**				0.722
≤60	94 (48.0)	52	42	
>60	102 (52.0)	59	43	
**Gender**				0.526
Male	95 (48.5)	56	39	
Female	101 (51.5)	55	46	
**Smoking**				0.683
Never	126 (64.3)	70	56	
Ever	70 (35.7)	41	29	
**Pleural invasion**				0.005
No	141 (71.9)	71	70	
Yes	55 (28.1)	40	15	
**Tumor length (cm)**				0.046
≤4	97 (49.5)	47	42	
>4	99 (50.5)	64	43	
**Location**				0.677
Central	49 (25.0)	29	20	
Peripheral	147 (75.0)	82	65	
**Differentiation**				0.024
Well or moderate	51 (26.0)	22	29	
Poor	145 (74.0)	89	56	
**T stage**				0.273
T1-T2	146 (74.5)	86	60	
T3-T4	50 (25.5)	25	25	
**N stage**				0.004
N0	26 (13.3)	8	18	
N1-N3	170 (86.7)	103	67	
**TNM stage**				0.001
I/II	97 (49.5)	66	31	
III	99 (50.5)	45	54	
**Treatment**				
Surgery only	25 (12.8)	7	18	0.000
Surgery with chemotherapy	102 (52.0)	71	31	
Surgery with chemoradiotherapy	69 (35.2)	33	36	

**Table 4:** Univariate and multivariate analyses of the prognostic factors in 196 patients with lung adenocarcinoma

	Univariate analysis			Multivariate analysis		
	*p* value	HR	95%CI	*p* value	HR	95%CI
Age (≤60 years vs >60 years)	0.177	1.276	0.895-1.818			
Gender (female vs male)	0.958	0.991	0.697-1.408			
Smoking (ever vs never)	0.524	1.126	0.781-1.624			
Pleural invasion (yes vs no)	<0.001	2.024	1.398-2.930	0.297	1.235	0.831-1.836
Differentiation (well/moderate vs poor)	<0.001	2.586	1.614-4.141	0.248	1.345	0.814-2.222
Tumor length (≤4 cm vs >4 cm)	<0.001	2.051	1.428-2.947	0.508	1.167	0.739-1.842
Location (central vs peripheral)	0.264	0.799	0.538-1.185			
T stage (T1/T2 vs T3/T4)	0.005	1.725	1.176-2.531	0.063	0.617	0.371-1.026
N stage (N0 vs N1/N2/N3)	<0.001	8.172	3.010-22.185	0.015	3.618	1.281-10.217
TNM stage (I/II vs III)	<0.001	3.250	2.234-4.727	<0.001	3.662	2.261-5.931
LIMCH1 (High vs Low)	0.024	0.660	0.460-0.947	0.005	0.545	0.358-0.830

**Table 5:** Gene Set Enrichment Analysis of LIMCH1 in lung adenocarcinoma

NAME	ES	NES	NOM p-value	FDR q-value
HALLMARK_MTORC1_SIGNALING	0.530	1.942	0.002	0.0813
HALLMARK_MYC_TARGETS_V1	0.601	1.914	0.022	0.052
HALLMARK_MYC_TARGETS_V2	0.711	1.893	0.006	0.042
HALLMARK_DNA_REPAIR	0.487	1.881	0.004	0.035
HALLMARK_E2F_TARGETS	0.710	1.835	0.012	0.0472
HALLMARK_G2M_CHECKPOINT	0.619	1.701	0.044	0.118
HALLMARK_GLYCOLYSIS	0.381	1.537	0.038	0.192
HALLMARK_UV_RESPONSE_UP	0.331	1.459	0.049	0.220

## Abbreviations

LUAD: lung adenocarcinoma
LUSC: lung squamous carcinoma
IHC: immunohistochemistry
NSCLC: non-small lung cancer
TCGA: The Cancer Genome Atlas
GEO: Gene Expression Omnibus
GSEA: gene set enrichment analysis
FDR: false discovery rate
TKIs: tyrosine kinase inhibitor
HR: hazard ratio
95%CI: 95% confidence interval
ROC: receiver operating characteristic
AUC: area under the curve
Espin: ESPN
ESCC: esophageal squamous cell carcinoma
TWF1: Twinfilin-1
NM-II: non-muscle myosin II
WHO: World Health Organization's
TMA: tissue microarray
DAB: diaminobenzidine
IRS: immunoreactive scoring
TNM stage: tumor-node-metastasis stage
